# Immune landscape of neoadjuvant chemoradiotherapy: involvement of MAL, a T-cell differentiation protein

**DOI:** 10.32604/or.2025.063419

**Published:** 2025-06-26

**Authors:** KOSEI NAKAJIMA, YOSHINORI INO

**Affiliations:** 1 Division of Molecular Pathology, National Cancer Center Research Institute, 5-1-1 Tsukiji, Chuo-ku, 104-0045, Tokyo Japan; 2 Division of Translational Research, Exploratory Oncology Research & Clinical Trial Center, National Cancer Center, 5-1-1 Tsukiji, Chuo-ku, 104-0045, Tokyo Japan; 3 Division of Surgery, Faculty of Veterinary Medicine, Imabari Campus, Okayama University of Science, 1-3 Ikoinooka, Imabari, 794-8555, Ehime Japan

**Keywords:** Neoadjuvant/preoperative therapy (NAT), Anti-tumor immunity, T-cell differentiation

## Abstract

**Background:**

Neoadjuvant/preoperative therapy (NAT) involves the administration of chemotherapy, with or without radiation, prior to surgical resection. This approach is commonly used for locally advanced tumors to reduce tumor volume, improve resectability, and minimize the need for extensive surgical procedures. While NAT has been shown to be effective in inducing local anti-tumor immunity in potentially resectable solid tumors, the underlying molecular mechanisms remain poorly understood.

**Methods:**

Cohort samples from pancreatic cancer patients who underwent NAT (n = 26) and those who did not (n = 20) were analyzed. Changes in the immune microenvironment induced by NAT were assessed using stratified bioinformatic approaches, including heatmap analysis of immune-related genes selected via Gene Ontology, Gene Set Enrichment Analysis (GSEA) with the immunologic signature database, and Ingenuity Pathway Analysis (IPA). Findings were further validated through immunohistochemical analysis.

**Results:**

A comprehensive, stratified evaluation integrating pathological and bioinformatic approaches revealed that NAT induced the upregulation of 212 genes, including DC-SIGN (CD209), and activated 13 immune-associated pathways, such as T-cell receptor (TCR) signaling. Additionally, NAT promoted an increased shift toward CD8 (+) T-cell populations through the upregulation of MAL (T-cell differentiation protein). Immunohistochemical analysis further confirmed a significant accumulation of DC-SIGN (+) dendritic cells and MAL (+) lymphocytes in NAT-treated patients.

**Conclusions:**

NAT enhances anti-tumor immunity by promoting CD8 (+) T-cell generation through the activation of DC-SIGN (+) dendritic cells and MAL (+) lymphocytes. This study is the first to report an increase in MAL (+) lymphocytes following NAT. Given its potential significance, further investigation in other solid tumors treated with NAT is warranted.

## Introduction

Pancreatic cancer remains one of the most challenging malignancies, characterized by poor prognosis and limited therapeutic efficacy. Survival rates vary considerably depending on the stage at diagnosis, including localized, regional, and distant stages [1]. Standard treatment modalities encompass surgical resection, radiation therapy, and chemotherapy, as well as emerging approaches such as targeted therapy and immunotherapy [2]. Despite its aggressive nature, ongoing research and the development of novel therapeutic strategies continue to offer promise for improved survival outcomes and enhanced quality of life in affected patients.

Neoadjuvant/preoperative therapy (NAT) involves the administration of chemotherapeutic agents with or without radiation before surgical resection [3]. Treatments are often offered for locally advanced lesions, aiming to shrink the tumor volume, increase surgical resectability, and reduce the need for extensive procedures. Thus, NAT is effective in locally advanced yet potentially resectable solid tumors, particularly in pancreatic, esophageal, gastric, rectal, breast, lung, bladder, and head and neck cancers, as well as soft tissue sarcoma and osteosarcoma or others. In addition, accumulating evidence from various tumors suggests that NAT also offers an immunological advantage [4] by inducing immunogenic cell death (ICD) by exposing calreticulin and releasing ATP, type 1 interferon, and other damage-associated molecules [5,6]. Subsequently, these chemoradiotherapy-induced events recruit intratumoral dendritic cells, leading to the presence of cancer-associated antigens, and an increase in the number of infiltrating lymphocytes [7–9].

Clinical guidelines from the National Comprehensive Cancer Network (NCCN), American Society of Clinical Oncology (ASCO), and European Society for Medical Oncology (ESMO) endorse NAT for borderline resectable and locally advanced pancreatic cancer to improve resectability and patient outcomes [10–12]. NAT consists of S-1, Gemcitabine, nab-paclitaxel, and a FOLFIRINOX regimen (folinic acid, fluorouracil, irinotecan plus oxaliplatin combination) with or without radiation [13,14]. This treatment has been reported to modulate the immune microenvironment by histopathology-based analysis, which showed an increasing number of CD4, CD8, MDSC, and MHC class1 A/B(+) cells and a decrease in the number of regulatory T cells [15–19] The precise mechanism remains unclear because comprehensive analysis to delineate molecular profiles induced by NAT has been limited and is only available in other cancers. Intriguingly, immunological changes in local lesions treated with chemoradiotherapy may affect and regress metastatic lesions, a phenomenon known as the abscopal effect. Because pancreatic cancer has been shown to have a dire prognosis due to its potent distant metastasis rate and highly invasive properties to surrounding tissues, an in-depth analysis of immunological changes caused by NAT could be a beneficial finding to halt the incurable disease.

In this study, we identified a novel mechanism underlying treatment-driven immune response in the pancreatic cancer microenvironment. NAT induces a shift toward CD8 (+) T-cell generation via the DC-SIGN-MAL axis, leading to enhanced antitumor immunity.

## Materials & Methods

### Patients and samples

This study was approved by the Institutional Review Board of the National Cancer Center, Tokyo, Japan, #2016-006 for biobank and #2005-077 for clinical (subsequent extension was granted in 2016 until 2024). Informed consent was obtained in writing from all participants involved in this study, and all clinical investigations were conducted according to the principles of the Declaration of Helsinki. All patients were subjected to standard clinical practice. Clinical and pathological data were obtained through a detailed retrospective review of the medical records of all 26 patients with ductal adenocarcinoma of the pancreas who had undergone NAT followed by surgical resection between April 2009 and March 2017 at the National Cancer Center Hospital, Tokyo, Japan. 13 patients were male and 13 were female, with a mean age of 63.5 years (range, 45–80 years). NAT included S-1 (n = 10), gemcitabine (n = 9), gemcitabine plus S-1 (n = 5), gemcitabine plus nab-paclitaxel (n = 2), SIROX (S-1, irinotecan plus oxaliplatin, n = 1), and FOLFIRINOX (folinic acid, fluorouracil, irinotecan plus oxaliplatin, n = 8) with radiation (n = 6). The median period after initiation of NAT to surgery was 8.4 months (range: 2.8–35.2 months). The median response rate was 36.1% (range: 2.5%–66.7%). Of these, 53.8% of patients showed a partial response (n = 14), 26.9% of patients showed a stable disease (n = 7) based on RECIST (Response Evaluation Criteria in Solid Tumors) criteria, and five patients were unable to be evaluated. Surgically resected specimens were fixed in 10% formalin, embedded in paraffin, and then cut into serial 5-mm-thick slices. Subsequently, the tissue sections were stained with hematoxylin and eosin (HE) for pathological examination and confirmed as pancreatic ductal adenocarcinomas.

### Microarray analysis

Frozen tumor tissues were embedded in OCT compound and stored at −80°C until use. Frozen sections were sliced and RNA was extracted using RNeasy Mini kits (Qiagen, 74104, Hilden, Germany). The RNA integrity number (RIN) was evaluated using a 2100 Bioanalyzer (Agilent, G2939A, Santa Clara, CA), and the values were all confirmed to be >8.0. Microarray analysis was performed at the Chemical Evaluation Research Institute (CERI, Tokyo, Japan). Briefly, Agilent’s One-Color Microarray-Based Gene Expression Analysis Low Input Quick Amp Labeling ver. 6.9, 100 ng of total RNA was used to generate Cy3-labeled cRNA. Subsequently, the samples were hybridized on a SurePrint G3 Human GE 8 × 60 K Microarray ver. 3.0 (Agilent, G4851C). The arrays were scanned using a DNA Microarray Scanner (Agilent, G2565CA) and the acquired images were quantified using Feature Extraction ver. 10.7.1.1 (Agilent, G4460-90053). Signals were normalized using GeneSpring GX 14.5 software (Agilent). To compare the two groups of samples, i.e., pancreatic cancer tissue with or without NAT, Welch’s *t*-test was used to determine statistical significance (*p* < 0.05).

For Ingenuity Pathway Analysis (IPA), statistically significant genes were classified into upregulated or downregulated gene groups. The data were then separately imported into the IPA software ver. 43605602 (Qiagen), and canonical pathway analysis was performed by the core analysis program.

For heat map analysis, hierarchical clustering of gene expression data was performed after molecular classification using Gene Ontology [20]. For immune-associated molecules, a gene list was created based on GO:0006954 (GO term: inflammatory response) and GO:0002376 (GO term: immune system process). In total. 3252 immunological genes were evaluated for their expression status.

Using GSEA software (GSEA 3.0, Broad Institute, Cambridge, MA), gene expression profiling in the immune microenvironment was performed using the Immunologic Signature database [21], which enables a detailed analysis of transcriptomic data for further biological understanding of immune processes. Briefly, it is an annotated compendium of approximately 5000 gene sets established by numerous published studies in human and mouse immunology. The detailed contents of each gene set are available online at the following site; http://software.broadinstitute.org/gsea/msigdb/genesets.jsp?collection=C7 (accessed 27 April 2025).

## Immunohistochemistry

Immunohistochemistry was performed on formalin-fixed, paraffin-embedded tissue sections using the avidin-biotin complex method. For antigen retrieval, tissue sections were autoclaved at 121°C for 10 min in the appropriate buffers, i.e., Envision^TM^ FLEX Target Retrieval Solution high or low pH (Agilent, GV80511-2 and GV80911-2, respectively) or Histofine simple stain kit (Nichirei, 414322, Tokyo, Japan) or a citrate buffer (10 mM, pH 6.0; Muto Pure Chemicals, 80159, Tokyo, Japan). For double-fluorescence immunohistochemistry, tissue sections were first blocked with 2% normal swine serum (JR Scientific, 44035, Woodland, CA, USA) in PBS for 1 h. Subsequently, the sections were incubated overnight at 4°C with primary antibodies against DC-SIGN and Siglec-1 (CD169). The following day, endogenous autofluorescence was quenched using the Vector TrueVIEW Autofluorescence Quenching Kit with DAPI (Vector Laboratories, Inc., SP-8500, Newark, CA) for 5 min. Sections were then incubated with the appropriate Alexa Fluor 488- (anti-mouse for DC-SIGN antibody) and Alexa Fluor 594-(anti-rabbit for Siglec-1 antibody) conjugated secondary antibodies (Thermo Fisher Scientific Inc., A-11001 and A-21207, respectively, Waltham, MA, USA) for 1 h. Nuclear counterstaining was performed using DAPI (Vector TrueVIEW Autofluorescence Quenching Kit with DAPI, Vector Laboratories, Inc., SP-8500, Newark, CA, USA) for 10 min (commercially prepared reagent containing pre-mixed DAPI was utilized in this study), followed by mounting with an anti-fade mounting medium (Vector Laboratories, Inc., H-1700). The staining procedure was optimized for each antibody. Consequently, antibody titration and antigen retrieval were the most appropriate methods, as summarized in Supplementary Table 1. For quantitative assessment of the immunohistochemical results for each molecule, slide images were scanned using a NanoZoomer 2.0-HT (Hamamatsu Photonics, S210, Hamamatsu, Japan), and the stained area and number of positive cells were analyzed using Tissue Studio software (Definiens, Munich, Germany) to minimize interobserver variance. DAB (3,3′-diaminobenzidine) and HistoGreen (Linaris Biologische Produkt, E109, Dossenheim, Germany) were used as chromogens for double immunohistochemistry as previously described [3,4]. MAL- and T-bet-positive cells were manually counted because of the staining of other cellular structures in addition to immune cells.

### Statistical analysis

For quantification of immunohistochemistry data, the nonparametric Mann–Whitney test was used to evaluate the statistical significance for the comparison of two categorical groups, i.e., pancreatic cancer patients with or without NAT. Differences were considered statistically significant at *p* < 0.05. Statistical analyses were performed using the IBM SPSS Statistics version 25.0 software (SPSS, Chicago, IL, USA).

## Results

### Neoadjuvant/preoperative chemoradiation stimulates immunological pathways

**Figure 1:**
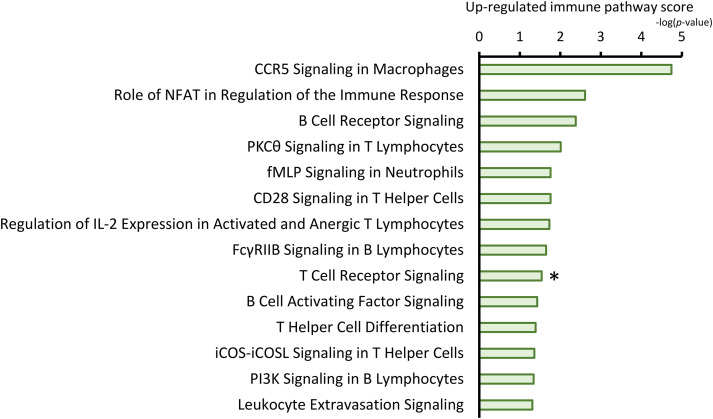
Neoadjuvant/preoperative treatment (NAT)-induced pathways associated with the immune response. Ingenuity pathway analysis (IPA) unveiled the most significantly associated immune pathways from the clusters of upregulated genes following NAT. For statistical analysis, the Welch’s *t*-test was used to compare the difference with/without NAT. Then, the pathways were ranked from the lowest (top) to the highest (bottom) *p*-values. The green bars indicate the log(*p*-value) of each pathway; therefore, the length of the bars represents the strength of the statistical association. *, focused pathway.

We first examined how therapeutic intervention*s*, i.e., preoperative/neoadjuvant chemoradiotherapy (NAT), alter the immune microenvironment in clinical settings. To analyze the therapeutic effects, we prepared two pairs of frozen samples collected from patients undergoing upfront surgery (not received NAT) or conversion surgery (received NAT). The microarray dataset was analyzed using Ingenuity Pathway Analysis (IPA) software to delineate the most significantly associated pathways from the clusters of upregulated genes by NAT. The results showed that 106 pathways were upregulated in patients who received NAT of statistical significance (*p* < 0.05). Among them, the following 14 pathways associated with immune responses (Fig. 1): CCR5 signaling in macrophages (*p* < 0.0001), the role of NFAT in the regulation of the immune response (*p* = 0.0025), B cell receptor signaling (*p* = 0.0042), PKCθ signaling in T lymphocytes (*p* = 0.0098), fMLP signaling in neutrophils (*p* = 0.0174), CD28 signaling in T helper cells (*p* = 0.0174), regulation of IL-2 expression in activated and anergic T lymphocytes (*p* = 0.0186), FcγRIIB Signaling in B Lymphocytes (*p* = 0.0223), T cell receptor signaling (*p* = 0.0288), B cell activating factor signaling (*p* = 0.0372), T helper cell differentiation (*p* = 0.0407), iCOS-iCOSL signaling in T helper cells (*p* = 0.0437), PI3K signaling in B lymphocytes (*p* = 0.0457), and leukocyte extravasation signaling (*p* = 0.0490). The results indicated that NAT significantly impacts immunological pathways in the pancreatic cancer microenvironment.

### Neoadjuvant/preoperative chemoradiation induces DC-SIGN-positive dendritic cells

**Figure 2:**
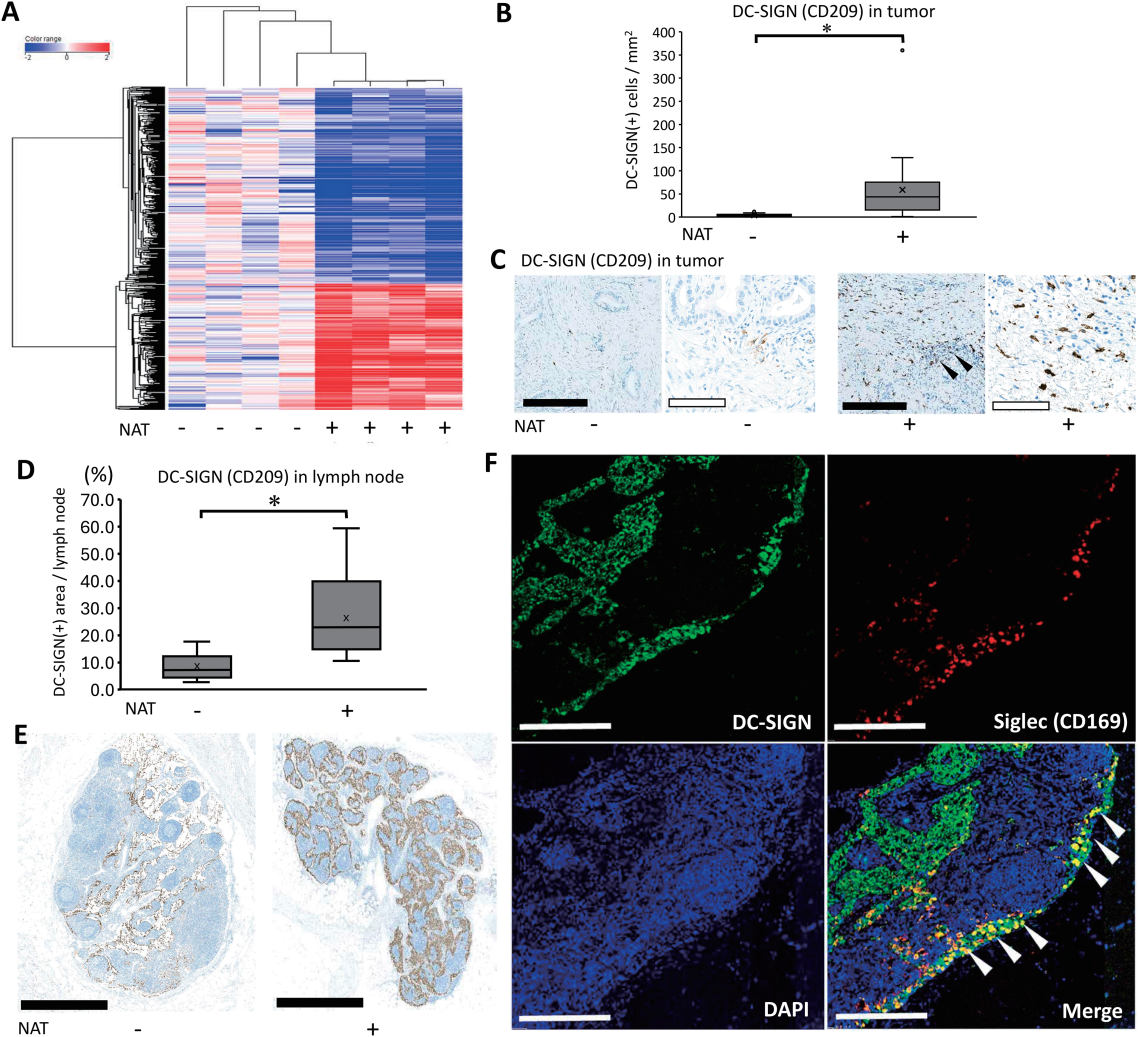
Neoadjuvant/preoperative chemoradiation induces DC-SIGN-positive dendritic cells. **(A)** Heat map analyses delineated a dynamic alteration of gene expression profile using Neoadjuvant/preoperative chemoradiotherapy (NAT) in immune-associated molecules. Based on the result of heatmap analysis, CD209 (DC-SIGN), a dendritic cell marker, was selected for further immunohistochemical evaluation. **(B)** DC-SIGN (+) cell numbers in tumor tissue were quantified by an automatic image analyzer for objective evaluation. For statistical analysis, the Mann–Whitney test was used to compare the difference between the pathological samples from patients with upfront surgery (not received NAT, n = 20) and conversion surgery (received NAT, n = 26) in tumor tissue. The data was unpaired (i.e., belonging to different patients) *, Differences with *p* < 0.05 were considered statistically significant. **(C)** Immunohistochemical staining of DC-SIGN using tumor tissue. Pathological samples from patients with upfront surgery (not received NAT) or conversion surgery (received NAT) in tumor tissue were compared. The black bar indicates 250 μm, and the white bar indicates 100 μm. Black arrowheads indicate residual cancer foci. **(D)** DC-SIGN (+) cell numbers in adjacent lymph nodes were quantified by an automatic image analyzer for objective evaluation. For statistical analysis, the Mann–Whitney test was used to compare the difference between the pathological samples from patients with upfront surgery (not received NAT, n = 13) and conversion surgery (received NAT, n = 14) in the adjacent lymph node. The data was unpaired (i.e., belonging to different patients) *, Differences with *p* < 0.05 were considered statistically significant. **(E)** Immunohistochemical staining of DC-SIGN using adjacent lymph node. Pathological samples from patients with upfront surgery (not received NAT) or conversion surgery (received NAT) in adjacent lymph nodes were compared. The black bar indicates 1 mm. **(F)** Fluorescent immunohistochemistry by detection of DC-SIGN and CD169. White arrowheads indicate that double-positive cells of DC-SIGN (+) and CD169 (+) were identified at the boundary of the adjacent lymph node sinus. White bars indicate 250 μm.

To further visualize the immunological effects of preoperative/neoadjuvant chemoradiotherapy (NAT), we selected immune-related genes annotated by Gene Ontology [20] and generated a heat map including genes with statistically significant changes (Fig. 2A). The results showed that 212 genes were upregulated (Supplementary Table 2) and 318 genes were downregulated. Consistent with the pathway analysis, the results indicated that NAT induced significantly altered immune-related molecules in the pancreatic cancer microenvironment. Dendritic cells serve as pivotal mediators of effective anti-tumor immunity, as demonstrated by their essential roles in antigen presentation and subsequent T-cell activation [22]. Among the listed genes, dendritic cell/macrophage-related molecules were specifically upregulated such as CLEC4G (LSECtin), S100A12, and CD209 (DC-SIGN). Therefore, it was necessary to detect the localization of these three molecules using immunohistochemistry. The findings indicated that S100A12 was detected in monocytes and macrophages in blood vessels and cancerous tissues. LSECtin was detected in dendritic cells in the lymph nodes, but not in the cancer stroma. Some cancer foci were positive for LSECtin after the NAT (Supplementary Fig. 1). DC-SIGN (+) cells were identified on dendritic cells in both the lymph nodes and cancer stroma (Fig. 2B–E). Previous reports have shown that DC-SIGN of dendritic cells plays crucial roles in anti-cancer immunity, specifically in the initial contact between dendritic cells and resting T lymphocytes [23] therefore, we focused on DC-SIGN (+) dendritic cells. We then histologically examined how DC-SIGN (+) cells changed after NAT using pathological slides collected from patients with or without NAT. The number of positive cells was quantified using an automatic image analyzer for objective evaluation. The results indicated that DC-SIGN (+) cells significantly increased in the cohort that received NAT in tumor tissue (Fig. 2B,C). Additionally, we observed a higher abundance of DC-SIGN (+) dendritic cells in the lymph nodes of the NAT group compared with the untreated group (*p* = 0.0000364) (Fig. 2B). We further evaluated the maturation status of dendritic cells by measuring the expression of Langerin (CD207) for immature dendritic cells and DC-LAMP (CD208) for mature dendritic cells. The results showed that the number of Langerin (CD207) (+) cells was not changed by NAT (*p* = 0.580), whereas the number of DC-LAMP (CD208) (+) cells tended to increase; however, this difference was not statistically significant (*p* = 0.268) (Supplementary Fig. 2). These data show that dendritic cells are likely to play an additional immunologic role by expressing DC-SIGN, independent of their maturation status. Next, we examined the DC-SIGN (+) area of the adjacent lymph node. We observed that there was a significant increase in DC-SIGN (+) dendric cells in the lymph nodes of the NAT group compared with the untreated group (Fig. 2D,E). To further explore the role of DC-SIGN (+) dendritic cells, we attempted to detect Siglec (CD169), a crucial molecule for the presentation of tumor-derived antigens, initiating early activation of tumor antigen-specific CD8 (+) T cells [24,25]. Immunohistochemistry results showed that DC-SIGN (+) and CD169 (+) double-positive dendritic cells were minor populations in the tumor lesions (data not shown), but many were primarily localized within the lymphatic sinus, an interstitial space functioning as a lymphatic conduit. This observation suggests their migration between the tumor and the lymph nodes (Fig. 2F). Collectively, NAT dynamically induces DC-SIGN (+) cells in tumor lesions, and a part of the dendritic population may migrate into the draining lymph nodes, where they express Siglec to activate lymphocytes.

### Neoadjuvant/Preoperative chemoradiation confers an increasing shift of the CD8 T-cell population

**Figure 3:**
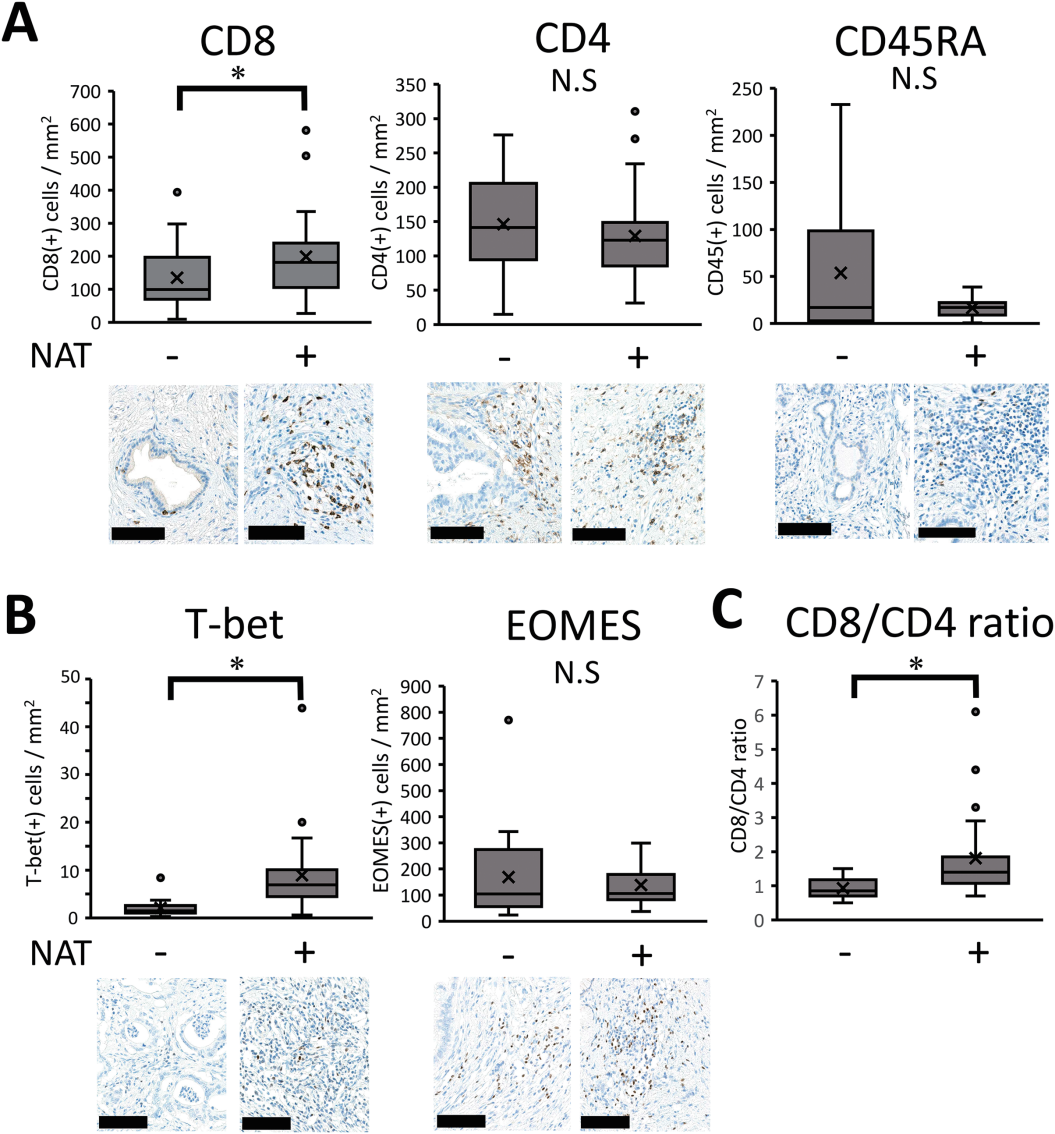
Immunohistochemical results of lymphocyte-associated molecules. **(A–C)** Pathological samples from patients with upfront surgery (not received NAT, n = 20) or conversion surgery (received NAT, n = 26) were compared. Then, the positive cell numbers were quantified by an automatic image analyzer for the purpose of objective evaluation. For statistical analysis, the Mann–Whitney test was used to compare the difference with/without NAT. The data was unpaired (i.e., belonging to different patients) *, Differences at *p* < 0.05 were considered statistically significant. N.S., not significant. The black bar indicates 100 μm.

To comprehensively assess immunological changes induced by neoadjuvant/preoperative chemoradiation (NAT), we employed GSEA analysis with an immunological signature database established by a massive accumulation of previously published studies in human and mouse immunology [21], which enabled us to analyze a detailed understanding of treatment-driven immune responses. The results showed that 63 immunological gene sets were highly elevated, with statistical significance (normalized *p*-value < 0.05). Among these gene sets showing high enrichment scores, we focused ‘GSE22886_CD8_VS_CD4_NAIVE_TCELL_DN,’ indicating an increasing shift of CD8 T-cells compared to CD4 T-cells with altered naïve lymphocyte population (Supplementary Fig. 3, asterisk). Based on the bioinformatics analysis, we performed immunohistochemistry to examine how CD4 (+) T cells, CD8 (+) T cells, and naïve lymphocytes respond to NAT. The positive cell numbers were quantified using an automatic image analyzer. The results indicated that the number of CD8 (+) cells significantly increased after NAT. In contrast, CD4 (+) and CD45RA (+) naïve cells tended to be reduced by NAT, but the differences were not statistically significant (*p* = 0.535 and *p* = 0.465, respectively) (Fig. 3A). To further clarify the lymphocyte lineage, we examined the transcription factors of lymphocytes, such as T-bet for Th1 cells, GATA-3 for Th2 cells, ROR-t for Th17 cells [26], and EOMES for CD8 cells [27]. The number of T-bet (+) T cells significantly increased in patients who received NAT *p* < 0.001) (Fig. 3B) whereas GATA-3 and ROR-γt (+) lymphocytes were both minor populations in the pancreatic cancer microenvironment; therefore, it was difficult to compare the histological changes (data not shown). There was no significant difference in EOMES (+) lymphocytes between the two patient cohorts (*p* = 0.825) (Fig. 3B). Next, to analyze the immunological shift between CD4 (+) and CD8 (+) cells, we calculated and statistically examined the ratio of CD8 (+) to CD4 (+) cells. The results showed that the CD8/CD4 ratio increased in patients who received NAT (*p* = 0.001) (Fig. 3C), indicating a shift in the CD8 (+) T cell population compared to CD4 (+) T cells, consistent with the GSEA analysis.

### MAL, T-cell differentiation protein, may be responsible for CD8 T-cell shift

**Figure 4:**
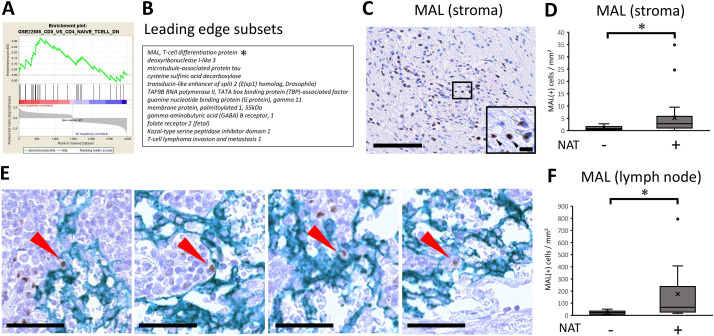
MAL, T-cell differentiation protein, may be responsible for CD8 T-cell shift. **(A)** GSEA Enrichment plot for a selected gene set depicting an immune response. Gene set enrichment analysis (GSEA) was performed with the Immunologic Signature database [21]. The green curve corresponds to the ES (enrichment score) curve, which is the running sum of the weighted enrichment score obtained from GSEA software. Signal-to-noise ratio (SNR) statistics were used to rank the genes per their correlation with either the immunological signature by receiving neoadjuvant/preoperative chemoradiotherapy (NAT) (Neo: Red) or the signature by patients not received NAT (PK: Blue). *, The asterisk denotes the molecule most relevant in the analysis. **(B)** The leading-edge subset indicates the core group of genes that accounts for the gene set’s enrichment signal. The table shows 13 leading-edge genes to be responsible for the enrichment plot. **(C)** Immunohistochemical analysis of expression of MAL, T-cell differentiation protein. Note that MAL expression is located on lymphoid cells. The black bar indicates 250 μm. (Inset) There were two expression patterns, either cytoplasmic localization (white arrowhead) or nuclear translocation (black arrowhead). The black bar indicates 10 μm. **(D)** With regards to the positive lymphoid cells of MAL expression, pathological samples from patients with upfront surgery (not received NAT, n = 20) or conversion surgery (received NAT, n = 26) were compared. For statistical analysis, the Mann–Whitney test was used to compare the difference with/without NAT. The data was unpaired (i.e., belonged to different patients) *, Differences at *p* < 0.05 were considered statistically significant. **(E)** In the draining lymph nodes, the interaction between MAL (+) lymphocytes and DC-SIGN (+) dendritic cells was observed (Red arrows). The black bar indicates 50 μm. **(F)** In the draining lymph nodes, MAL (+) lymphocytes were increased in patients with NAT. For statistical analysis, the Mann–Whitney test was used to compare the difference with/without NAT. The data was unpaired (i.e., belonged to different patients) *, Differences at *p* < 0.05 were considered statistically significant.

The immunological shift prompted us to investigate the molecular mechanism(s) underlying the immune microenvironment, and we further examined the molecules responsible for this shift (Fig. 4A). The enrichment plot of the GSEA analysis revealed the responsible molecules for the gene set, termed ‘leading edge subsets’. The subset consisted of 13 molecules that contributed to the CD8 (+) T cell shift (Fig. 4B). Among the listed molecules, it was necessary to focus on MAL, a T-cell differentiation protein, because MAL has been implicated to be required for activation of T-cell receptor (TCR) signaling, inducing T-cell differentiation [28,29]. In this study, pathway analysis also showed that TCR signaling was highly upregulated by NAT (Fig. 1); therefore, the targeted molecule, MAL, was considered a possible candidate. We then histologically examined how MAL (+) cells changed after the NAT. The result showed that MAL was detected in the lymphoid cells of the cancer stroma (Fig. 4C). Notably, there were two expression patterns, cytoplasmic or nuclear (Fig. 4C, inset). The number of MAL (+) lymphocytes significantly increased in patients who received NAT (Fig. 4D), indicating that MAL may be responsible for the CD8 (+) T-cell shift induced by NAT. Additionally, MAL (+) lymphocytes were found in the draining lymph nodes, with some expressing ICAM-3, a DC-SIGN-binding protein (Supplementary Fig. 4A). MAL (+) lymphocytes interacted with DC-SIGN (+) dendritic cells in the lymph nodes (Fig. 4E). Similarly to the results for cancer stroma, the number of MAL (+) lymphocytes in draining lymph nodes significantly increased in patients who received NAT (Fig. 4F). Next, we examined the cellular features of MAL (+) lymphocytes and stained them for several T-cell and differentiation markers, such as CD3, CD4, CD8, EOMES, T-bet, and CD45RA. The results showed that some CD3 (+) T lymphocytes expressed MAL, indicating that the MAL (+) lymphocytes were of T-cell origin. However, MAL (+) lymphocytes did not co-stain with either CD4 or CD8. In addition, MAL is often co-localized with CD45RA, EOMES, and T-bet (Supplementary Fig. 4B). These results indicate that the MAL (+) lymphocytes were in the intermediate differentiation stage of the T-cell lineage expressing transcription factors that induce CD8 differentiation or Th1 response, contributing to antitumor immunity by NAT in pancreatic cancer.

## Discussion

The data presented here provides comprehensive insights into the dynamic changes in the immune microenvironment following neoadjuvant therapy (NAT) in pancreatic cancer. This study is the first to report an increase in MAL (+) lymphocytes following NAT. Given its potential significance, further investigation is warranted in other solid tumors where NAT is utilized, including esophageal, gastric, rectal, breast, lung, and bladder cancers, as well as head and neck cancers, soft tissue sarcomas, and osteosarcomas.

Pancreatic cancer foci act as a protective barrier against immune cells, being encapsulated by dense collagen layers that impede immune responses. NAT may improve the immune environment in pancreatic cancer, where a low number of immune cells is not likely to trigger a strong immune response. This treatment reduced collagen synthesis in cancer-associated fibroblasts via Ephrin-A signaling. Subsequently, types 1, 3, and 4 collagen volume in the tumor mass tended to decrease in patients who received NAT, as reported in our previous study [3]. The reduction of collagen could enhance immune cell infiltration into tumor tissue by the following events: NAT to treat pancreatic cancer, specifically oxaliplatin and radiation, potently induce immunogenic cell death (ICD) and scattering tumor-associated antigens, which may recruit intratumoral DC-SIGN (+) dendritic cells, which possess promising antigen presentation potential. Although the immunological changes in draining lymph nodes by NAT have been limited, as shown, some of the DC-SIGN (+) dendritic cells may migrate into draining lymph nodes, whereby they express Siglec, present tumor-derived antigens, and initiate the activation of tumor antigen-specific CD8 (+) T cells. The extrathymically developed CD8 (+) T cells have been implicated in immune responses against tumor cells. DC-SIGN was originally identified as a dendritic cell-specific adhesion receptor that interacts with resting lymphocytes, leading to enhanced proliferation of T cells at the initiation of the immune response [23]. In the tumor microenvironment, DC-SIGN (+) dendritic cells mediate efficient tumor-associated antigen presentation to T lymphocytes via ICAM-3, a DC-SIGN-binding protein that leads to the regression of established tumors [30–32]. Some CD3 (+) T lymphocyte populations express MAL, a T-cell differentiation protein that interacts with lymphocyte-specific protein tyrosine kinase (Lck), a crucial molecule for the activation of T-cell receptor (TCR) signaling, leading to the activation of T-cells [28,29]. MAL then translocates to the nucleus and may contribute to transcriptional activation [33]. Also, MAL controls protein sorting at the supramolecular activation cluster (SMAC)/immunological synapse (IS) of T lymphocytes [34] These events may increase the number of T-bet and Th1 cell marker-positive lymphocytes, thereby inducing a Th1 response, leading to CD8 (+) cytotoxic T cell activation, partially by a previous study [35]. As such, NAT for pancreatic cancer induces a shift toward CD8 (+) T-cell generation via the DC-SIGN-MAL axis, leading to enhanced anti-tumor immunity. Although it remains uncertain whether MAL-associated TCR-signaling T cells specifically differentiate into CD8 (+) T cells, in the immune response against cancer cells, TCR signaling has been reported to preferentially activate and promote the proliferation of CD8 (+) T cells [36,37]. The precise mechanism of differentiation is not fully elucidated, and further analyses are needed.

**Figure 5:**
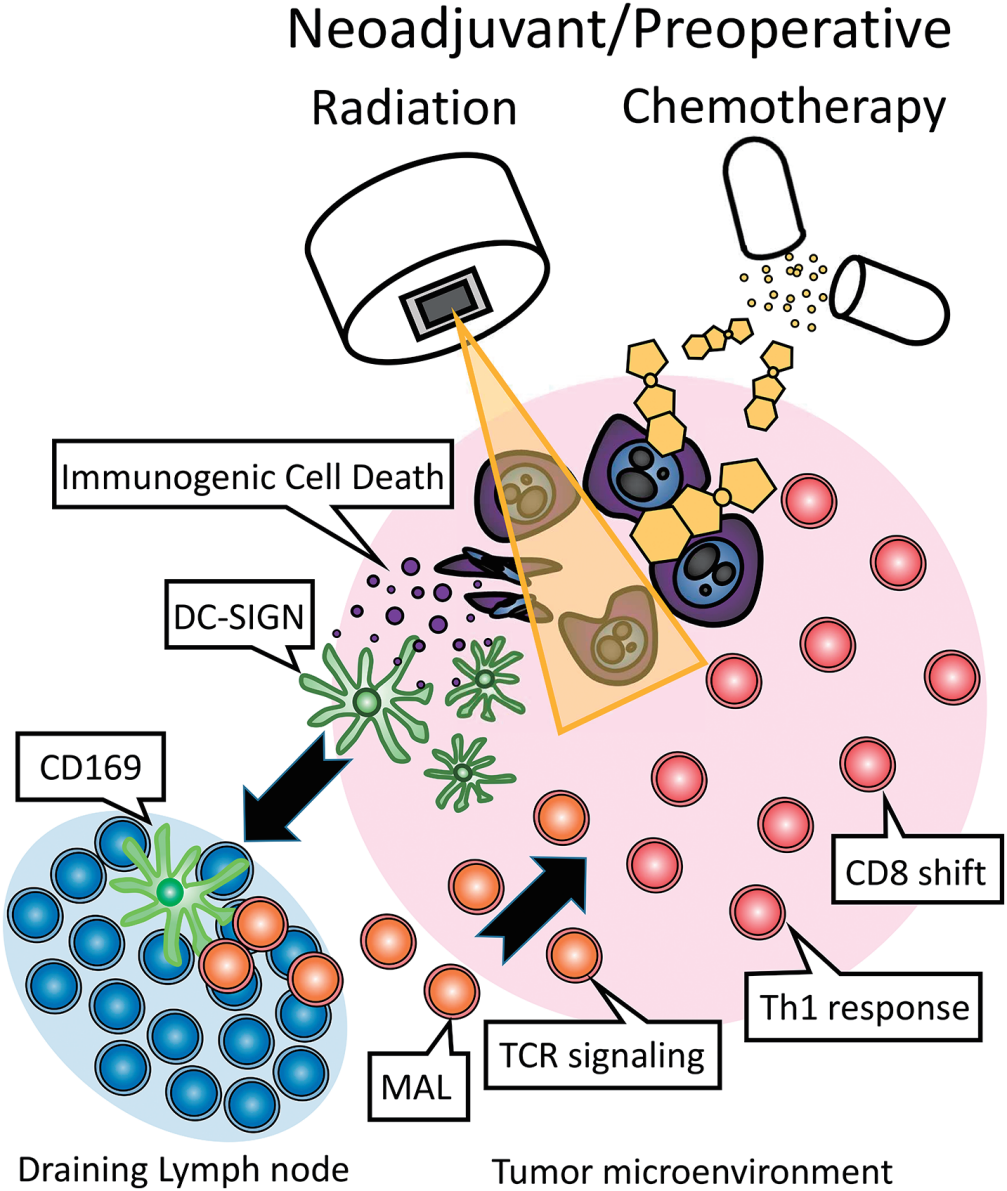
The proposed immunological effects of neoadjuvant/preoperative chemoradiation (NAT) in the treatment of pancreatic cancer involve the induction of immunogenic cell death (ICD) by chemotherapy and/or radiation. This process facilitates the recruitment of intra-tumoral DC-SIGN (+) dendritic cells, a subset of which migrates to the draining lymph nodes. Within the lymph nodes, these dendritic cells upregulate CD169 (Siglec), thereby activating lymphocytes. This activation leads to the expression of MAL, which in turn enhances TCR signaling, promotes a Th1 immune response, and drives the proliferation of CD8 T cells.

In this study, we presented a possible mechanism by which NAT affects the immune system. The proposed immunological effect of NAT has been delineated (Fig. 5). Briefly, NAT induces immunogenic cell death (ICD), promoting the recruitment of intra-tumoral DC-SIGN (+) dendritic cells. A subset migrates to draining lymph nodes, where they upregulate CD169 (Siglec), activating lymphocytes. This activation enhances MAL expression, augments TCR signaling, induces a Th1 response, and drives CD8 T cell proliferation. Thereafter, it may also be possible to induce systemic spread of the immune response, as demonstrated in previous studies on other tumors. These endogenous immune enhancements may be part of the underlying mechanism by which NAT prolongs the overall survival of patients with pancreatic cancer [38]. Specifically, a recent randomized controlled trial showed that NAT followed by surgery improved overall survival compared with upfront surgery and adjuvant chemotherapy in pancreatic cancer [39]. The clinical significance of this study is to clarify the possible immune mechanisms underlying prolonged overall survival. Exogenous immunotherapy may intensify this effect.

Recent advancements in liquid biopsy technologies have greatly improved cancer diagnostics and treatment by providing non-invasive methods for detecting and monitoring malignancies, as demonstrated in our clinical studies [40]. As shown in Supplementary Table 2, NAT led to the upregulation of multiple immunomodulatory factors, including IL-6 (147.5-fold), CCL2 (17.1-fold), BMP6 (6.4-fold), IL-33 (5.0-fold), CXCL12 (4.7-fold), IGF1 (3.5-fold), IL-10 (2.5-fold), TNFSF8 (2.0-fold), CX3CL1 (1.9-fold), PDGF-D (1.7-fold), CCL16 (1.6-fold), and TNFSF12 (1.4-fold), thereby influencing the immunological tumor microenvironment. Notably, IL-6 and CCL2, which exhibited the highest fold increases, are known to suppress cytotoxic CD8 (+) T-cell function, while MAL upregulation has also been implicated in immune modulation. Given these findings, further investigation is warranted to determine whether these immunosuppressive mechanisms can be effectively detected and monitored using liquid biopsy techniques.

Regarding study limitations, the influence of patient characteristics (age, sex, and disease stage) and adjacent (non-cancerous) tissue were not considered in this study. As for the sample size, the analyses were performed using a small cohort, which might limit the drawing of proper conclusions from the observed changes. Also, sample size may affect the study results as seen in a previous study [3]. The small cohort size is due to the challenge of obtaining fresh pancreatic cancer samples after effective NAT. Effective NAT changes the tumor to be hard to detect the tumor shape and its position of the fresh surgical specimen macroscopically on the cut surface when fresh tumor samples are obtained. Effective NAT also changes the hard mass that is a characteristic of pancreatic cancer to a vague mass by palpation. Such changes made it hard to take fresh tumor samples. The bioinformatic methodological flow also has certain limitations, as the selection of screened genes may introduce potential biases. Given that this study focuses on immunological changes in the tumor microenvironment following NAT, we prioritized the selection of molecules related to dendritic cells and macrophages. However, this targeted approach may inadvertently overlook other potentially significant factors due to the exclusion of background noise signals from various genes. Therefore, as demonstrated in this study, a stratified and comprehensive evaluation strategy for immunological responses can help mitigate the risk of overlooking unconsidered factors. Integrating pathological and bioinformatic approaches is essential to obtaining accurate and robust evidence. As for the other molecules in the tumor microenvironment such as fibroblasts and collagens, we have divided the several groups of crucial molecules in the tumor microenvironment based on Gene Ontology and then analyzed [3]. Also, to correctly evaluate the cytological response of immune cells to NAT, it is necessary to evaluate additional experimental verification methods, such as flow cytometry and western blotting. Indeed, we have tried to examine using these methods from patient’s samples surgically removed but the results were unstable when initial and preliminary experiments (data not shown). The reason is that the pancreatic cancer microenvironment is characterized by very thick and hard collagen layers, so they did not allow us to obtain stable results by these molecular and cytological detections. Thus, a comprehensive approach combining pathology and bioinformatics was used in this study.

## Conclusion

In conclusion, our study demonstrates that NAT induces a shift toward CD8 (+) T-cell generation via the DC-SIGN-MAL axis, leading to enhanced anti-tumor immunity. The pathological evaluation of MAL (+) lymphocytes after surgical resection following NAT for borderline resectable and locally advanced pancreatic cancer may serve as an indicator of the immunological anti-tumor response, providing potential clinical applications for patient outcomes.

## Supplementary Materials

Supplementary Figure 1 **Immunohistochemical screening results of dendritic cell/macrophage-related molecules**. **(A)** S100A12 expression in cancer stroma, lymph node, and blood vessel. The black arrowheads indicate the blood vessels. **(B)** CLEC4G (LSECtin) expression on dendritic cells which is located in cancer stroma, lymph node. Also, the granular expression patterns on cancer foci were noted after NAT. The black arrowheads indicate the cancer foci. Black bars indicate 250 μm.

Supplementary Figure 2 **The CD207 (Langerin) and CD208 (DC-LAMP) were measured to analyze the maturation status of dendritic cells**. For statistical analysis, the Mann–Whitney test was used to compare the difference with/without NAT. The data was unpaired (i.e. belong to different patients) N.S., not significant. Black bars indicate 250 μm.

Supplementary Figure 3 **GSEA analysis listed the top 30 immunologic gene sets with high enrichment scores and statistical significance (normalized *p*-value < 0.05)**. The pathways were ranked from lowest *p*-values (up) to higher (down). The blue bars indicate -log(*p*-value) of each pathway, so the length of the bars represents the strength of the statistical association. The detailed contexts of each gene set are available on the online site: http://software.broadinstitute.org/gsea/msigdb/genesets.jsp?collection=C7. *, focused gene set.

Supplementary Figure 4 **Immunohistochemical characterization of MAL (+) Lymphocytes**. **(A)** MAL (+) lymphocytes also expressed ICAM3 in lymph nodes. Brown color indicates MAL expression, whereas dark green indicates co-expression of MAL and ICAM3 (white arrows). Black bars indicate 50 μm. **(B)** Double immunohistochemistry results were performed for clarification of immunological features of MAL (+) lymphocytes. The dark green indicates co-expression of MAL and one of the focused lymphocyte-related molecules of interest i.e.CD3, CD4, CD8, CD45RA, Tbet, and EOMES. Brown color indicates MAL expression, whereas green color indicates single expression of focused lymphocyte-related molecules. The white arrows indicate that MAL (+) lymphocytes often co-expressed with CD45RA, EOMES, and T-bet. Black bars indicate 50 μm.

## Data Availability

The datasets used and analyzed in the current study are available from the corresponding author upon reasonable request.

## Supplementary Materials

The supplementary material is available online at https://www.techscience.com/doi/10.32604/or.2025.063419/s1.
