# Supplementary figures and images for: Immune landscape of neoadjuvant chemoradiotherapy: involvement of MAL, a T-cell differentiation protein
Source: Oncol Res. 2025 Jun 26;33(7):1769–79. doi: 10.32604/or.2025.063419 (PMC12215608; doi:10.32604/or.2025.063419)

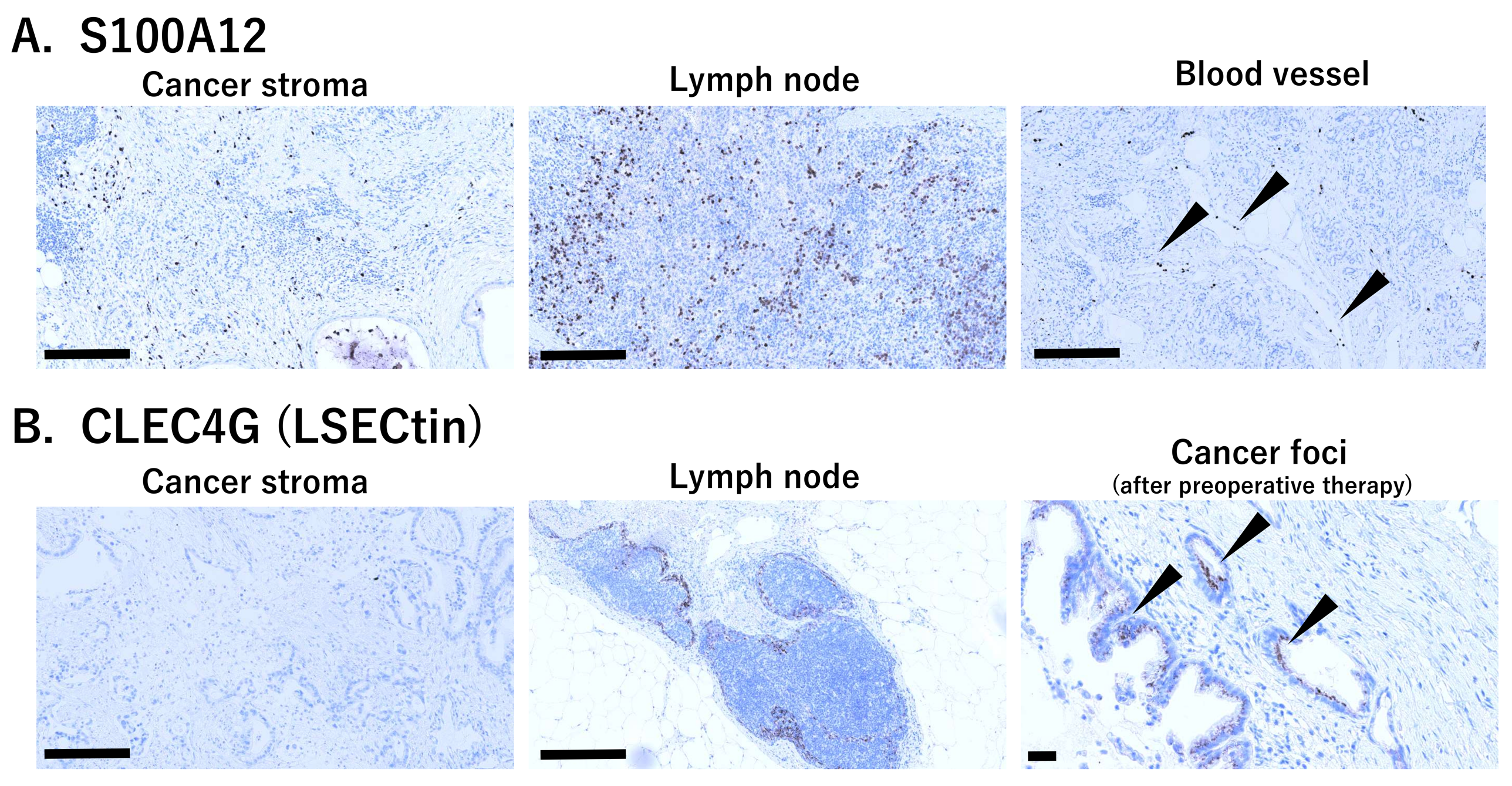

Supplement: Supplementary Figure 1 [file OncolRes-33-63419-s001.tif]

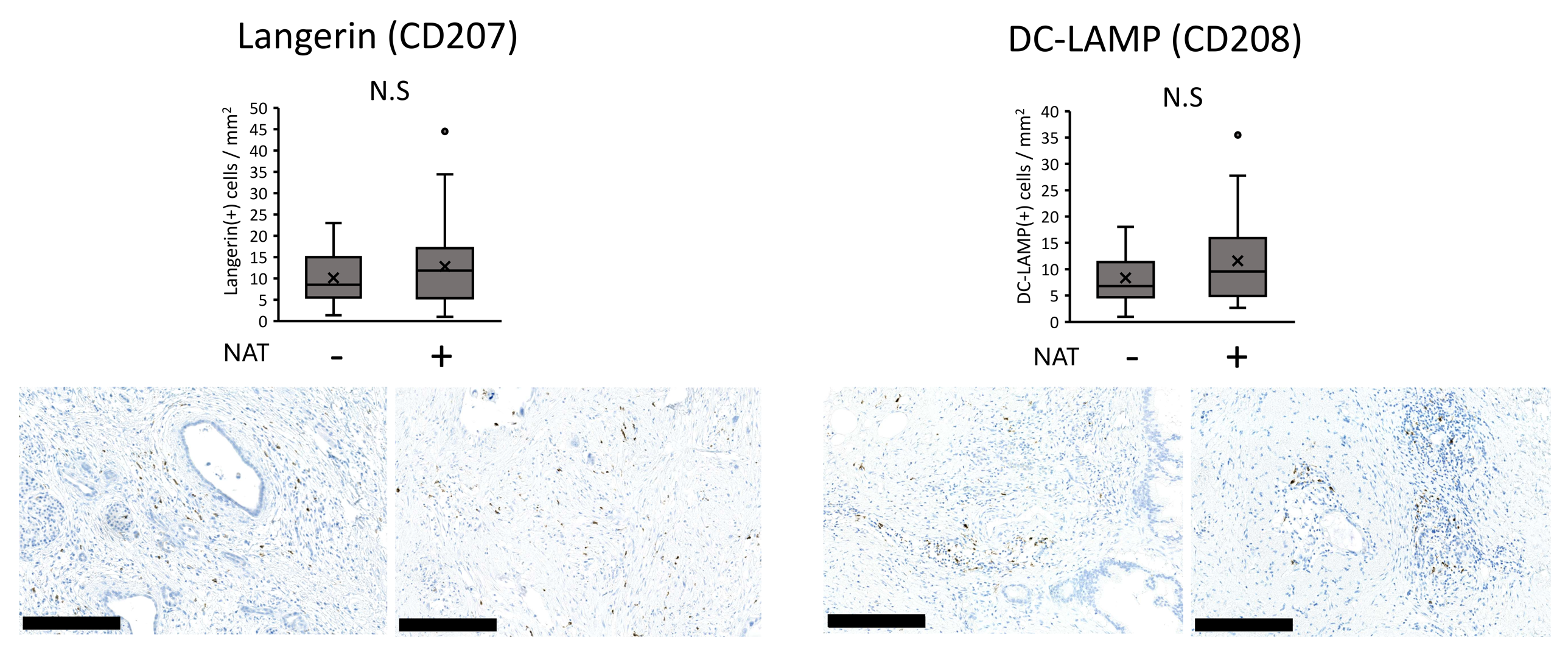

Supplement: Supplementary Figure 2 [file OncolRes-33-63419-s002.tif]

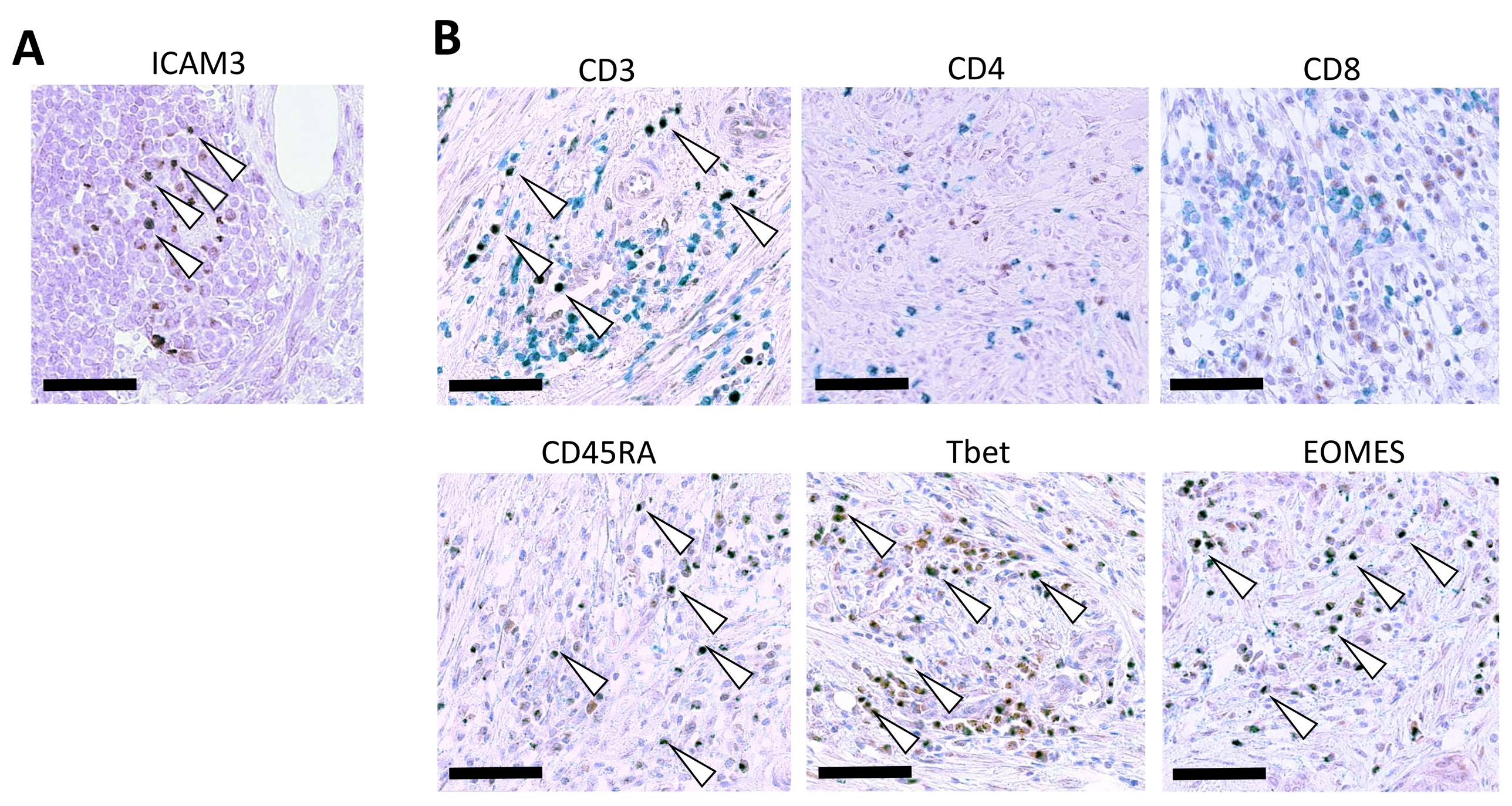

Supplement: Supplementary Figure 4 [file OncolRes-33-63419-s004.tif]
