# Supplementary material for: Immune landscape of neoadjuvant chemoradiotherapy: involvement of MAL, a T-cell differentiation protein
Source: Oncol Res. 2025 Jun 26;33(7):1769–79. doi: 10.32604/or.2025.063419 (PMC12215608; doi:10.32604/or.2025.063419)
Supplement: Supplementary file 5 [file OncolRes-33-63419-s005.docx]

**Supplementary Table 1: Immunohistochemical staining condition.**

| Antigen | Company | Species | Catalogue Number | Antigen retrieval | Dilution |
| --- | --- | --- | --- | --- | --- |
| CD3 | Abcam  (Cambridge, UK) | rabbit, monoclonal | ab16669 | Envision TM FLEX Target Retrieval Solution (Low pH) | x100 |
| CD4 | BioSB  (SantaBarbara, CA) | rabbit, monoclonal | BSB5150 | citrate buffer | ready to use |
| CD8 | Leica (Wetzlar, Germany) | mouse, monoclonal | NCL-L-CD8-4B11 | Histofine | x50 |
| CD45RA | Dako  (Santa Clara, CA ) | mouse, monoclonal | M0754 | citrate buffer | x500 |
| T-bet | santa cruz  (Santa Cruz, CA) | mouse, monoclonal | sc-21749 | citrate buffer | x50 |
| EOMES | invitrogen  (Waltham, CA) | mouse, monoclonal | 14-4877-82 | citrate buffer | x200 |
| ROR-gt | invitrogen | rat, monoclonal | 14-6988-82 | citrate buffer | x100, with CSA-II |
| GATA-3 | santa cruz | mouse, monoclonal | sc-268 | citrate buffer | x50 |
| DC-SIGN (CD209) | santa cruz | mouse, monoclonal | sc-65740 | citrate buffer | x100 |
| DC-LAMP (CD208) | R&D systems  (Minneapolis, MN) | goat, polyclonal | AF4087 | citrate buffer | x500 |
| Langerin (CD207) | Leica | mouse, monoclonal | NCL-L-LANGERIN | Envision TM FLEX Target Retrieval Solution (Low pH) | x50 |
| Siglec-1 (CD169) | spring bioscience  (Pleasanton, CA) | rabbit, monoclonal | sc-53442 | citrate buffer | x100 |
| MAL (T-cell differentiation protein) | santa cruz | mouse, monoclonal | sc-390687 | Envision TM FLEX Target Retrieval Solution (Low pH) | x250 |
| LSECtin (CLEC4G) | Abcam | rabbit, monoclonal | ab181196 | Envision TM FLEX Target Retrieval Solution (Low pH) | x100 |
| S100A12 | Sigma | rabbit, polyclonal | HPA002881 | citrate buffer | x100 |
| ICAM3 | Abcam | rabbit, monoclonal | ab109505 | Envision TM FLEX Target Retrieval Solution (Low pH) | x500 |
| Alexa Fluor 488-congugated secondary antibody | Thermo Fisher Scientific (Waltham, MA) | Goat anti-mouse IgG (H+L) for detection of DC-SIGN primary antibody | A-11001 | - | x200 |
| Alexa Fluor 594-congugated secondary antibody | Thermo Fisher Scientific | anti-rabbit IgG (H+L) for detection of Siglec primary antibody | A-21207 | - | x200 |
| biotinylated  secondary antibody | Vector laboratories | anti-mouse IgG (H+L) | BA-2000 | - | x200 |
| biotinylated  secondary antibody | Vector laboratories | anti-rabbit IgG (H+L) | BA-1000 | - | x200 |
| biotinylated  secondary antibody | Vector laboratories | anti-rat IgG (H+L) | BA-9400-1.5 | - | x200 |
| biotinylated  secondary antibody | Vector laboratories | anti-goat IgG (H+L) | BA-9500-1.5 | - | x200 |

The antibodies used for immunohistochemistry are listed below. The antigen retrieval methods and dilutions are also shown.

**Supplementary Table 2: A list of immune-associated genes upregulated by neoadjuvant/preoperative chemoradiotherapy (NAT) is presented.**

| Probe # | Expression fold_neo/pk | Gene symbol | Gene Name |
| --- | --- | --- | --- |
| A_23_P369237 | 171.3 | ADIPOQ | adiponectin, C1Q and collagen domain containing |
| A_23_P71037 | 147.5 | IL6 | interleukin 6 |
| A_23_P121064 | 107.4 | PTX3 | pentraxin 3, long |
| A_23_P203558 | 49.6 | HBB | hemoglobin, beta |
| A_23_P398566 | 42.3 | NR4A3 | nuclear receptor subfamily 4, group A, member 3 |
| A_23_P321949 | 28.1 | PLA2G2A | phospholipase A2, group IIA (platelets, synovial fluid) |
| A_23_P153390 | 22.7 | CLEC4G | C-type lectin domain family 4, member G |
| A_23_P74001 | 22.4 | S100A12 | S100 calcium binding protein A12 |
| A_33_P3385785 | 20.5 | S100A12 | S100 calcium binding protein A12 |
| A_33_P3213235 | 19.4 | CLEC4G | C-type lectin domain family 4, member G |
| A_23_P74059 | 19.4 | NPPA | natriuretic peptide A |
| A_23_P89431 | 17.1 | CCL2 | chemokine (C-C motif) ligand 2 |
| A_33_P3259708 | 10.7 | CMA1 | chymase 1, mast cell |
| A_24_P305345 | 8.5 | CD209 | CD209 molecule * |
| A_23_P207058 | 8.4 | SOCS3 | suppressor of cytokine signaling 3 |
| A_23_P216225 | 8.3 | EGR3 | early growth response 3 |
| A_23_P4773 | 8.3 | LILRB5 | leukocyte immunoglobulin-like receptor, subfamily B (with TM and ITIM domains), member 5 |
| A_24_P186539 | 7.6 | CD209 | CD209 molecule* |
| A_33_P3234202 | 7.5 | DNASE1L3 | deoxyribonuclease I-like 3 |
| A_23_P170649 | 7.0 | SBSPON | somatomedin B and thrombospondin, type 1 domain containing |
| A_23_P214080 | 6.6 | EGR1 | early growth response 1 |
| A_23_P19624 | 6.4 | BMP6 | bone morphogenetic protein 6 |
| A_23_P10121 | 6.3 | SFRP1 | secreted frizzled-related protein 1 |
| A_23_P32253 | 6.1 | NFIL3 | nuclear factor, interleukin 3 regulated |
| A_23_P119562 | 6.0 | CFD | complement factor D (adipsin) |
| A_33_P3401753 | 5.8 | FFAR3 | free fatty acid receptor 3 |
| A_23_P111583 | 5.8 | CD36 | CD36 molecule (thrombospondin receptor) |
| A_24_P363745 | 5.7 | LRRC14 | leucine rich repeat containing 14 |
| A_33_P3298810 | 5.6 | FFAR3 | free fatty acid receptor 3 |
| A_23_P389897 | 5.5 | NGFR | nerve growth factor receptor |
| A_23_P86470 | 5.4 | CH25H | cholesterol 25-hydroxylase |
| A_23_P39237 | 5.3 | ZFP36 | ZFP36 ring finger protein |
| A_23_P140384 | 5.1 | CTSG | cathepsin G |
| A_24_P89457 | 5.0 | CDKN1A | cyclin-dependent kinase inhibitor 1A (p21, Cip1) |
| A_23_P31945 | 5.0 | IL33 | interleukin 33 |
| A_33_P3352578 | 4.9 | CLEC4D | C-type lectin domain family 4, member D |
| A_23_P91278 | 4.8 | NFATC2 | nuclear factor of activated T-cells, cytoplasmic, calcineurin-dependent 2 |
| A_23_P202448 | 4.7 | CXCL12 | chemokine (C-X-C motif) ligand 12 |
| A_23_P137697 | 4.5 | SELP | selectin P (granule membrane protein 140kDa, antigen CD62) |
| A_23_P253602 | 4.5 | BMX | BMX non-receptor tyrosine kinase |
| A_33_P3339100 | 4.5 | SELP | selectin P (granule membrane protein 140kDa, antigen CD62) |
| A_23_P141505 | 4.4 | CLEC10A | C-type lectin domain family 10, member A |
| A_23_P213857 | 4.4 | C7 | complement component 7 |
| A_24_P231104 | 4.2 | LEPR | leptin receptor |
| A_33_P3358183 | 4.1 | PTGFR | prostaglandin F receptor (FP) |
| A_33_P3227990 | 4.1 | MBP | myelin basic protein |
| A_23_P103765 | 4.0 | FCER1A | Fc fragment of IgE, high affinity I, receptor for; alpha polypeptide |
| A_23_P215913 | 3.9 | CLU | clusterin |
| A_33_P3316539 | 3.8 | SLC7A2 | solute carrier family 7 (cationic amino acid transporter, y+ system), member 2 |
| A_24_P31275 | 3.8 | ATP1B2 | ATPase, Na+/K+ transporting, beta 2 polypeptide |
| A_23_P22444 | 3.8 | CFP | complement factor properdin |
| A_23_P143526 | 3.7 | S100B | S100 calcium binding protein B |
| A_33_P3363804 | 3.7 | NCAM1 | neural cell adhesion molecule 1 |
| A_33_P3250939 | 3.6 | RAB3C | RAB3C, member RAS oncogene family |
| A_23_P28334 | 3.6 | IL18RAP | interleukin 18 receptor accessory protein |
| A_33_P3363799 | 3.6 | NCAM1 | neural cell adhesion molecule 1 |
| A_21_P0000171 | 3.5 | IL6R | interleukin 6 receptor |
| A_33_P3376551 | 3.5 | PTGFR | prostaglandin F receptor (FP) |
| A_23_P113572 | 3.5 | CD19 | CD19 molecule |
| A_24_P304423 | 3.5 | IGF1 | insulin-like growth factor 1 (somatomedin C) |
| A_24_P167642 | 3.5 | GCH1 | GTP cyclohydrolase 1 |
| A_24_P126139 | 3.5 | RAB9B | RAB9B, member RAS oncogene family |
| A_23_P13907 | 3.4 | IGF1 | insulin-like growth factor 1 (somatomedin C) |
| A_33_P3265744 | 3.4 | PTGER3 | prostaglandin E receptor 3 (subtype EP3) |
| A_24_P224488 | 3.4 | MAPT | microtubule-associated protein tau |
| A_23_P200780 | 3.4 | TGFBR3 | transforming growth factor, beta receptor III |
| A_33_P3414964 | 3.3 | PTPRS | protein tyrosine phosphatase, receptor type, S |
| A_23_P309739 | 3.3 | ESR1 | estrogen receptor 1 |
| A_33_P3712341 | 3.3 | CXCL12 | chemokine (C-X-C motif) ligand 12 |
| A_23_P8981 | 3.3 | STAR | steroidogenic acute regulatory protein |
| A_23_P214208 | 3.2 | CNR1 | cannabinoid receptor 1 (brain) |
| A_33_P3340639 | 3.2 | GPAM | glycerol-3-phosphate acyltransferase, mitochondrial |
| A_23_P106194 | 3.2 | FOS | FBJ murine osteosarcoma viral oncogene homolog |
| A_23_P401106 | 3.2 | PDE2A | phosphodiesterase 2A, cGMP-stimulated |
| A_33_P3221960 | 3.1 | IL18RAP | interleukin 18 receptor accessory protein |
| A_23_P89589 | 3.1 | PER1 | period circadian clock 1 |
| A_33_P3314559 | 3.1 | RAB3C | RAB3C, member RAS oncogene family |
| A_23_P142738 | 3.1 | TMEM178A | transmembrane protein 178A |
| A_23_P417891 | 3.1 | ARHGEF7 | Rho guanine nucleotide exchange factor (GEF) 7 |
| A_24_P32935 | 3.1 | FOLR2 | folate receptor 2 (fetal) |
| A_33_P3316522 | 3.1 | DEFB124 | defensin, beta 124 |
| A_23_P30736 | 3.1 | HLA-DOB | major histocompatibility complex, class II, DO beta |
| A_33_P3260342 | 3.0 | NFASC | neurofascin |
| A_23_P17345 | 2.9 | MAFB | v-maf avian musculoaponeurotic fibrosarcoma oncogene homolog B |
| A_23_P253317 | 2.9 | GPR171 | G protein-coupled receptor 171 |
| A_23_P406448 | 2.9 | RAB9B | RAB9B, member RAS oncogene family |
| A_23_P171074 | 2.9 | ITM2A | integral membrane protein 2A |
| A_33_P3254216 | 2.8 | MAPK10 | mitogen-activated protein kinase 10 |
| A_23_P47709 | 2.8 | FOLR2 | folate receptor 2 (fetal) |
| A_23_P128728 | 2.8 | ARG2 | arginase 2 |
| A_23_P376557 | 2.8 | MMP25 | matrix metallopeptidase 25 |
| A_33_P3326634 | 2.8 | GPC3 | glypican 3 |
| A_33_P3249976 | 2.7 | JAM2 | junctional adhesion molecule 2 |
| A_24_P370172 | 2.7 | LILRA5 | leukocyte immunoglobulin-like receptor, subfamily A (with TM domain), member 5 |
| A_33_P3376546 | 2.7 | PTGFR | prostaglandin F receptor (FP) |
| A_33_P3303449 | 2.7 | LEPR | leptin receptor |
| A_33_P3281695 | 2.6 | NLRP3 | NLR family, pyrin domain containing 3 |
| A_24_P201171 | 2.6 | STXBP1 | syntaxin binding protein 1 |
| A_33_P3288844 | 2.6 | IL6R | interleukin 6 receptor |
| A_33_P3265739 | 2.6 | PTGER3 | prostaglandin E receptor 3 (subtype EP3) |
| A_23_P45025 | 2.6 | MAPK10 | mitogen-activated protein kinase 10 |
| A_33_P3226212 | 2.6 | JAM2 | junctional adhesion molecule 2 |
| A_23_P30294 | 2.6 | CDO1 | cysteine dioxygenase type 1 |
| A_33_P3230264 | 2.6 | GPC3 | glypican 3 |
| A_23_P24433 | 2.6 | CTSF | cathepsin F |
| A_33_P3213374 | 2.5 | CITED2 | Cbp/p300-interacting transactivator, with Glu/Asp-rich carboxy-terminal domain, 2 |
| A_23_P208493 | 2.5 | LILRB2 | leukocyte immunoglobulin-like receptor, subfamily B (with TM and ITIM domains), member 2 |
| A_23_P8834 | 2.5 | EPHX2 | epoxide hydrolase 2, cytoplasmic |
| A_23_P119698 | 2.5 | CD320 | CD320 molecule |
| A_32_P223777 | 2.5 | IL6ST | interleukin 6 signal transducer |
| A_23_P126735 | 2.5 | IL10 | interleukin 10 |
| A_21_P0014031 | 2.4 | MAPT | microtubule-associated protein tau |
| A_21_P0000116 | 2.4 | MID1 | midline 1 |
| A_24_P128308 | 2.4 | KLF6 | Kruppel-like factor 6 |
| A_23_P122937 | 2.4 | ELMO1 | engulfment and cell motility 1 |
| A_23_P354151 | 2.3 | ITK | IL2-inducible T-cell kinase |
| A_33_P3262376 | 2.3 | OTUD7A | OTU deubiquitinase 7A |
| A_23_P348063 | 2.3 | SYNGR1 | synaptogyrin 1 |
| A_23_P41217 | 2.3 | CD200R1 | CD200 receptor 1 |
| A_23_P415021 | 2.3 | METTL7A | methyltransferase like 7A |
| A_33_P3352827 | 2.3 | SLAMF1 | signaling lymphocytic activation molecule family member 1 |
| A_33_P3406866 | 2.3 | NFASC | neurofascin |
| A_23_P214969 | 2.3 | CITED2 | Cbp/p300-interacting transactivator, with Glu/Asp-rich carboxy-terminal domain, 2 |
| A_23_P18246 | 2.3 | XCR1 | chemokine (C motif) receptor 1 |
| A_24_P412156 | 2.3 | CXCL12 | chemokine (C-X-C motif) ligand 12 |
| A_23_P420281 | 2.2 | PRKCB | protein kinase C, beta |
| A_23_P393777 | 2.2 | PTGDR | prostaglandin D2 receptor (DP) |
| A_23_P62647 | 2.2 | SLAMF1 | signaling lymphocytic activation molecule family member 1 |
| A_23_P137035 | 2.2 | PIR | pirin (iron-binding nuclear protein) |
| A_24_P178503 | 2.2 | ABCC9 | ATP-binding cassette, sub-family C (CFTR/MRP), member 9 |
| A_33_P3406873 | 2.2 | NFASC | neurofascin |
| A_33_P3294533 | 2.1 | PRKCB | protein kinase C, beta |
| A_23_P376686 | 2.1 | BTNL2 | butyrophilin-like 2 (MHC class II associated) |
| A_33_P3233834 | 2.1 | IL6ST | interleukin 6 signal transducer |
| A_33_P3247858 | 2.1 | MPP1 | membrane protein, palmitoylated 1, 55kDa |
| A_33_P3419460 | 2.1 | VAPA | VAMP (vesicle-associated membrane protein)-associated protein A, 33kDa |
| A_23_P12392 | 2.1 | PTPRC | protein tyrosine phosphatase, receptor type, C |
| A_23_P34045 | 2.1 | EDA | ectodysplasin A |
| A_32_P154342 | 2.1 | SLCO4C1 | solute carrier organic anion transporter family, member 4C1 |
| A_23_P99275 | 2.1 | KLRB1 | killer cell lectin-like receptor subfamily B, member 1 |
| A_23_P382199 | 2.1 | VAPA | VAMP (vesicle-associated membrane protein)-associated protein A, 33kDa |
| A_23_P201551 | 2.0 | VAV3 | vav 3 guanine nucleotide exchange factor |
| A_33_P3233843 | 2.0 | IL6ST | interleukin 6 signal transducer |
| A_23_P250564 | 2.0 | PRKCE | protein kinase C, epsilon |
| A_23_P169257 | 2.0 | TNFSF8 | tumor necrosis factor (ligand) superfamily, member 8 |
| A_23_P171296 | 2.0 | MPP1 | membrane protein, palmitoylated 1, 55kDa |
| A_33_P3352019 | 2.0 | SCARA3 | scavenger receptor class A, member 3 |
| A_33_P3225046 | 2.0 | CD34 | CD34 molecule |
| A_23_P23829 | 1.9 | CD34 | CD34 molecule |
| A_33_P3409508 | 1.9 | MAPK11 | mitogen-activated protein kinase 11 |
| A_32_P3914 | 1.9 | WIPF3 | WAS/WASL interacting protein family, member 3 |
| A_23_P120845 | 1.9 | XBP1 | X-box binding protein 1 |
| A_24_P208567 | 1.9 | IL18R1 | interleukin 18 receptor 1 |
| A_33_P3284404 | 1.9 | SYNGR1 | synaptogyrin 1 |
| A_33_P3380807 | 1.9 | TLR9 | toll-like receptor 9 |
| A_24_P324674 | 1.9 | LY9 | lymphocyte antigen 9 |
| A_33_P3379091 | 1.9 | SYNGR1 | synaptogyrin 1 |
| A_23_P500741 | 1.9 | CBFA2T3 | core-binding factor, runt domain, alpha subunit 2; translocated to, 3 |
| A_23_P211522 | 1.9 | SYNGR1 | synaptogyrin 1 |
| A_24_P390495 | 1.9 | CX3CL1 | chemokine (C-X3-C motif) ligand 1 |
| A_23_P378690 | 1.8 | TMEM64 | transmembrane protein 64 |
| A_23_P1473 | 1.8 | PRF1 | perforin 1 (pore forming protein) |
| A_23_P205746 | 1.8 | EML1 | echinoderm microtubule associated protein like 1 |
| A_23_P216023 | 1.8 | ANGPT1 | angiopoietin 1 |
| A_33_P3213493 | 1.8 | VAMP2 | vesicle-associated membrane protein 2 (synaptobrevin 2) |
| A_23_P57534 | 1.8 | DDX17 | DEAD (Asp-Glu-Ala-Asp) box helicase 17 |
| A_23_P207319 | 1.8 | MAP3K14 | mitogen-activated protein kinase kinase kinase 14 |
| A_21_P0014601 | 1.8 | SFPQ | splicing factor proline/glutamine-rich |
| A_23_P217384 | 1.7 | AP1S2 | adaptor-related protein complex 1, sigma 2 subunit |
| A_33_P3378514 | 1.7 | PDE5A | phosphodiesterase 5A, cGMP-specific |
| A_33_P3220837 | 1.7 | MAFB | v-maf avian musculoaponeurotic fibrosarcoma oncogene homolog B |
| A_24_P124349 | 1.7 | PDGFD | platelet derived growth factor D |
| A_24_P336551 | 1.7 | BGLAP | bone gamma-carboxyglutamate (gla) protein |
| A_24_P156769 | 1.7 | MPL | MPL proto-oncogene, thrombopoietin receptor |
| A_23_P39386 | 1.7 | HCST | hematopoietic cell signal transducer |
| A_23_P129358 | 1.7 | SETD6 | SET domain containing 6 |
| A_33_P3397399 | 1.7 | PPP3CB | protein phosphatase 3, catalytic subunit, beta isozyme |
| A_19_P00320158 | 1.7 | SEC14L1 | SEC14-like 1 (S. cerevisiae) |
| A_24_P174613 | 1.7 | FBXW7 | F-box and WD repeat domain containing 7, E3 ubiquitin protein ligase |
| A_33_P3413558 | 1.7 | CD226 | CD226 molecule |
| A_23_P106844 | 1.7 | MT2A | metallothionein 2A |
| A_23_P53126 | 1.7 | LMO2 | LIM domain only 2 (rhombotin-like 1) |
| A_23_P254079 | 1.7 | STBD1 | starch binding domain 1 |
| A_33_P3303572 | 1.7 | EDA | ectodysplasin A |
| A_23_P64661 | 1.7 | ARHGAP9 | Rho GTPase activating protein 9 |
| A_23_P253052 | 1.7 | CD99L2 | CD99 molecule-like 2 |
| A_24_P98524 | 1.7 | PPP3CB | protein phosphatase 3, catalytic subunit, beta isozyme |
| A_33_P3305482 | 1.7 | EIF2B3 | eukaryotic translation initiation factor 2B, subunit 3 gamma, 58kDa |
| A_23_P127964 | 1.7 | PRCP | prolylcarboxypeptidase (angiotensinase C) |
| A_33_P3258782 | 1.6 | AP1S2 | adaptor-related protein complex 1, sigma 2 subunit |
| A_23_P40847 | 1.6 | CHST2 | carbohydrate (N-acetylglucosamine-6-O) sulfotransferase 2 |
| A_33_P3292478 | 1.6 | CCL16 | chemokine (C-C motif) ligand 16 |
| A_23_P203743 | 1.6 | GAB2 | GRB2-associated binding protein 2 |
| A_33_P3409886 | 1.6 | VAMP2 | vesicle-associated membrane protein 2 (synaptobrevin 2) |
| A_33_P3296372 | 1.6 | EIF2B3 | eukaryotic translation initiation factor 2B, subunit 3 gamma, 58kDa |
| A_23_P62953 | 1.6 | PBX1 | pre-B-cell leukemia homeobox 1 |
| A_23_P47034 | 1.6 | HHEX | hematopoietically expressed homeobox |
| A_23_P59613 | 1.6 | FZD9 | frizzled class receptor 9 |
| A_23_P167920 | 1.6 | DLL1 | delta-like 1 (Drosophila) |
| A_23_P130169 | 1.6 | TBKBP1 | TBK1 binding protein 1 |
| A_24_P125283 | 1.6 | HDAC5 | histone deacetylase 5 |
| A_23_P22660 | 1.6 | CYSLTR1 | cysteinyl leukotriene receptor 1 |
| A_23_P207742 | 1.6 | THRA | thyroid hormone receptor, alpha |
| A_24_P313186 | 1.5 | CALM1 | calmodulin 1 (phosphorylase kinase, delta) |
| A_24_P171058 | 1.5 | TMEM64 | transmembrane protein 64 |
| A_24_P333733 | 1.5 | ATP6V0A1 | ATPase, H+ transporting, lysosomal V0 subunit a1 |
| A_23_P375566 | 1.5 | STXBP4 | syntaxin binding protein 4 |
| A_23_P208866 | 1.5 | GMFG | glia maturation factor, gamma |
| A_23_P164528 | 1.5 | WDR7 | WD repeat domain 7 |
| A_33_P3260445 | 1.4 | DYNLT1 | dynein, light chain, Tctex-type 1 |
| A_33_P3406623 | 1.4 | TNFSF12 | tumor necrosis factor (ligand) superfamily, member 12 |
| A_33_P3356406 | 1.2 | SOS1 | son of sevenless homolog 1 (Drosophila) |

In total, 212 genes were identified with statistical significance (*p*<0.05). The asterisks* indicated that the upregulation of CD209 (DC-SIGN) expression was confirmed using two different probes and was nominated as one of the candidate molecules.
